# Long-term trial of protection provided by adenovirus-vectored vaccine expressing the PPRV H protein

**DOI:** 10.1038/s41541-024-00892-2

**Published:** 2024-06-03

**Authors:** Karin E. Darpel, Amanda Corla, Anna Stedman, Fiona Bellamy, John Flannery, Paulina Rajko-Nenow, Claire Powers, Steve Wilson, Bryan Charleston, Michael D. Baron, Carrie Batten

**Affiliations:** 1 The Pirbright Institute, Ash Road, Pirbright, Surrey, GU24 0NF UK; 2 Institute of Virology and Immunology, Mittelhäusern, Switzerland; 3 Department of Infectious Diseases and Pathobiology, Vetsuisse Faculty, University of Bern, Bern, Switzerland; 4 APHA, Sand Hutton, York, YO41 1LZ Weybridge, UK; 5 Viral Vector Core Facility, Pandemic Sciences Institute, Oxford University, Oxford, UK; 6 Global Alliance for Livestock Veterinary Medicines, Edinburgh, UK; 7 Present Address: Veterinary Medicines Directorate, Woodham Lane, Addlestone, Surrey, KT15 3LS UK; 8 Present Address: Department of Pharmaceutical Sciences and Biotechnology, Technological University of the Shannon, Athlone, Ireland

**Keywords:** Vaccines, Infection

## Abstract

A recombinant, replication-defective, adenovirus-vectored vaccine expressing the H surface glycoprotein of peste des petits ruminants virus (PPRV) has previously been shown to protect goats from challenge with wild-type PPRV at up to 4 months post vaccination. Here, we present the results of a longer-term trial of the protection provided by such a vaccine, challenging animals at 6, 9, 12 and 15 months post vaccination. Vaccinated animals developed high levels of anti-PPRV H protein antibodies, which were virus-neutralising, and the level of these antibodies was maintained for the duration of the trial. The vaccinated animals were largely protected against overt clinical disease from the challenge virus. Although viral genome was intermittently detected in blood samples, nasal and/or ocular swabs of vaccinated goats post challenge, viral RNA levels were significantly lower compared to unvaccinated control animals and vaccinated goats did not appear to excrete live virus. This protection, like the antibody response, was maintained at the same level for at least 15 months after vaccination. In addition, we showed that animals that have been vaccinated with the adenovirus-based vaccine can be revaccinated with the same vaccine after 12 months and showed an increased anti-PPRV antibody response after this boost vaccination. Such vaccines, which provide a DIVA capability, would therefore be suitable for use when the current live attenuated PPRV vaccines are withdrawn at the end of the ongoing global PPR eradication campaign.

## Introduction

Peste des petits ruminants (PPR) is an economically important disease of livestock, primarily affecting sheep and goats. The disease is distributed through large parts of Asia, the entire Middle East and most countries in Africa north of Zimbabwe (see^1^ and references therein). Because of its significant economic impact, particularly in developing countries, the disease is the target of an ongoing global eradication campaign being co-ordinated by the World Organisation for Animal Health (WOAH) and the Food and Agriculture Organisation of the United Nations (FAO)^2^.

Control of PPR disease is primarily achieved by large scale vaccination, since movement control of livestock is largely not practicable in the countries where PPR is endemic. All the vaccines in use are live attenuated strains of PPR virus (PPRV), the virus that causes PPR^3,4^. These vaccines have proven themselves safe and effective over many years of use in the field, and can prevent disease caused by any known genetic lineage of PPRV^5^. Because these vaccines act essentially by causing a subclinical infection in the vaccinated animal, the antibody signature in vaccinated animals is identical to that in animals that have recovered from disease; at the moment, there is no test that can distinguish infected from vaccinated animals (DIVA test). Such a test is not absolutely required for the successful eradication of PPR, since the successful eradication of the equivalent disease of cattle, rinderpest, was accomplished without a DIVA test. Nevertheless, it is generally accepted that such a test, or a vaccine to facilitate such a test, would be very helpful in the closing stages of eradication when countries may wish to continue precautionary vaccination even when they have successfully cleared circulating wild-type virus from their own territories. In addition, if the same international protocols are applied following PPR eradication as were applied following rinderpest eradication, all forms of live PPRV will be proscribed, including the vaccine strains. A vaccine that is not itself live PPRV would be useful as an emergency response vaccine in case of re-emergence of the disease.

Several alternative vaccines against PPR have been published that would enable an effective DIVA test, including a genetically modified PPRV^6^, recombinant pox viruses^7,8^ and adenoviruses^9–12^ (reviewed in detail in^13^). In our own laboratory we have previously shown that a recombinant replication-defective human adenovirus type 5 (rAdV5) expressing the H surface glycoprotein of PPRV (rAdV5-H) is effective at protecting goats from experimental challenge with wild-type PPRV up to 15 weeks after vaccination, and would provide the possibility of a DIVA test^12,14^. Similar constructs have been published by other laboratories showing immunogenicity^9,10^ and protection from challenge with wild-type virus^11^. While it was clear from these studies that recombinant adenovirus-based vaccines showed promise as a DIVA vaccine, it was unknown how long protection could last, and whether it would be possible to revaccinate animals with the same vaccine, or if the effect of a second or subsequent vaccination would be blocked by a host response to adenovirus proteins. In this study we have carried out a longer-term study of the protection provided by rAdV5-H in goats, challenging vaccinated animals at 6, 9, 12 and 15 months post vaccination (mpv). We also revaccinated one group of animals at 12 mpv, and determined if such a booster vaccination could further stimulate anti-PPRV antibodies and enhance protection from challenge with wild-type virus. This experimental study therefore extends our knowledge of the duration of protection provided by a recombinant adenovirus-based vaccine to time points significantly greater than 9 months, and we show that, at least for the vaccine expressing the PPRV-H protein antigen, the immune response and level of protection are stable for at least 15 months. Furthermore we demonstrated that a booster vaccination at 12 months led to an increase in the titre of neutralising antibodies.

## Results

### Preliminary testing of challenge virus stocks

In order to be able to perform a valid comparison of the protection provided by the vaccine at different times post vaccination, it was necessary to have a stock of challenge virus that produced clear and unambiguous disease in experimental goats and that, stored at –80 °C, would provide a consistent challenge at each time after vaccination. Previous experience suggested that, while it is possible to passage wild-type PPRV in cell culture, not all preparations of the same isolate show the same pathogenicity^12,14,15^.

**Fig. 1:**
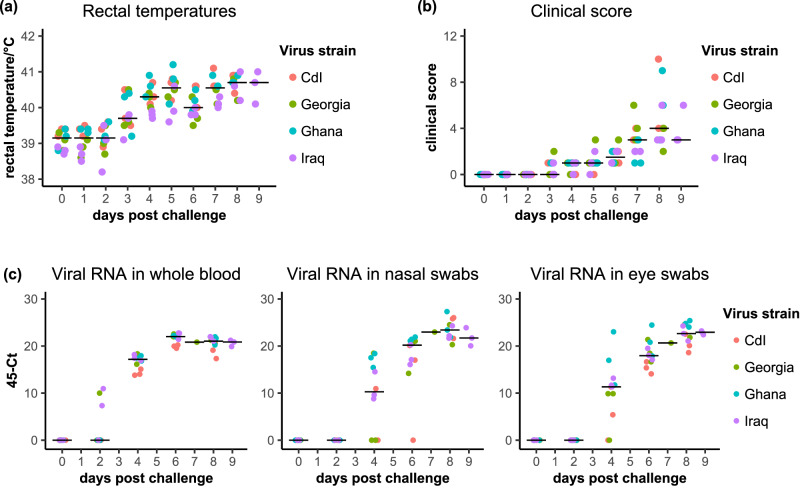
Testing of challenge virus stocks. Groups of goats (3 animals per group) were infected with PPRV/Cote d’Ivoire/1989 (CdI), PPRV/Georgia/2016 (Georgia), PPRV/Ghana/Accra/1978 (Ghana) or PPRV/Iraq/2011 (Iraq) and monitored for 8-9 days post challenge to establish the pattern of clinical disease elicited by the challenge. **a** The rectal temperatures of the infected animals (recorded daily); (**b**) the clinical score based on the scoring table as described in “Methods”; (**c**) viral RNA as quantitated using RT-qPCR on blood samples or swabs taken approximately every two days. The position of the median of all the samples at a particular time is indicated by a horizontal bar, and the data points are coloured according to the PPRV strain used to infect the animal.

We tested four preparations of wild-type PPRV: PPRV/Cote d’Ivoire/1989 (CdI), PPRV/Georgia/2016 (Georgia), PPRV/Ghana/Accra/1978 (Ghana) or PPRV/Iraq/2011 (Iraq). We used three animals per PPRV strain. Animals were infected with one of these strains by the nasal route and the animals monitored daily for clinical signs, including rectal temperature (Fig. 1a, b). Blood samples and ocular and nasal swabs were taken approximately every two days, more frequently as the animals reached peak disease, and the level of PPRV RNA in these samples determined by reverse transcription-real time PCR (RT-qPCR) (Fig. 1c). All of the infected animals developed overt and specific clinical signs of PPR, and the study was therefore terminated early for reaching its scientific endpoint. All the animals infected with the Ghana, Georgia and CdI strains were euthanised on day 8 (day 7 for one animal infected with the Georgia strain), while the animals infected with the Iraq strain were euthanised on day 9. There was no significant difference between the overall clinical scores for the four challenge strains. The pyrexia in the animals infected with the Iraq strain were significantly lower than those in the animals infected with the Ghana or CdI strains (*p* = 0.004 in each case), but there was no significant difference between the pattern of temperatures seen with the other three strains. There was no consistent difference in the levels of viral RNA seen in the blood, nasal swabs or ocular swabs taken from the animals in the different groups. No group had significantly different amounts of viral RNA in nasal swabs to those from any other group. In the ocular swab samples, animals infected with Ghana had significantly more viral RNA in these swabs than animals infected with CdI, but the swabs from neither of these groups of animals differed from those of the other two groups. Similarly, blood from animals infected with Iraq had more viral RNA than blood from animals infected with CdI, but neither of these two groups were significantly different to the other two.

Since all our virus stocks gave a similar, clear, disease profile, we opted to challenge with PPRV/Ghana/1978 simply because we had the largest stock of this batch of virus.

### Antibody responses to vaccination and duration of the response

**Fig. 2:**
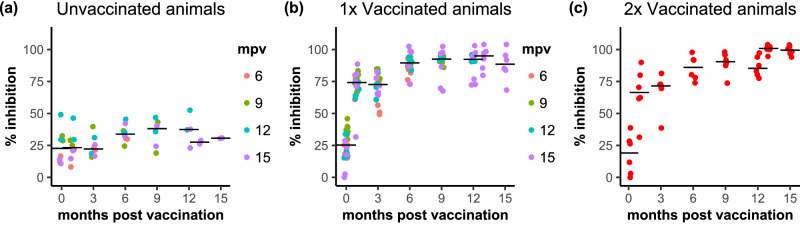
Induction of anti-PPRV H protein antibodies by the vaccine. Serum samples taken at the indicated times post vaccination were assayed using the PPRV-H protein specific cELISA and are shown for (**a**) the control unvaccinated animals, (**b**) the single (1x) vaccinated animals and (**c**) the animals vaccinated twice (2x). **a**, **b** Points are coloured according to the time post vaccination at which those animals were challenged. The position of the median of all the samples at a particular time is indicated by a horizontal bar.

**Fig. 3:**
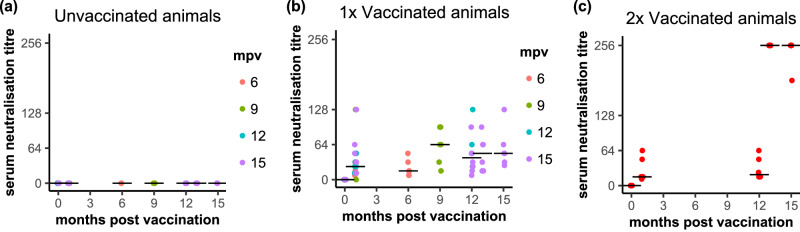
Induction of PPRV-neutralising antibodies by the vaccine. The titre of PPRV-neutralising antibodies was determined in serum samples taken at the indicated times post vaccination, as described in “Methods” and are shown for (**a**) the control unvaccinated animals, (**b**) the single (1x) vaccinated animals and (**c**) the animals vaccinated twice (2x). The results are expressed as the reciprocal of the titre dilution. For the unvaccinated and 1x vaccinated animals, the points are coloured according to the time post vaccination at which those animals were challenged. The position of the median value of all the samples at each time point is indicated by a horizontal bar.

The presence of anti-PPRV H protein antibodies was determined by competition ELISA (cELISA) for all experimental animals (vaccinated and non-vaccinated) at the start of the long-term vaccine study and at 1, 3 and 6 months post vaccination (mpv); those animals that had not yet been challenged with wild-type virus were further sampled and tested at 9, 12, 13 and 15 mpv (Fig. 2). All serum samples for any one animal were analysed at the same time on the same ELISA plate in order to avoid possible artefacts due to inter-assay variability. At 1 mpv, all vaccinated goats had significant levels of anti-H antibodies (% inhibition in the cELISA > 60%), and by 6 mpv the sera from all animals showed % inhibition >70%. This level was maintained over the course of the study. ANOVA analysis of data from 6 months onwards showed no change in the H antibody level in the single-vaccinated animals up to 15 mpv (i.e. mpv was not a significant factor in the linear model, *p* > 0.05). The second (booster) vaccination at 12 months increased the levels of anti-H antibody, with this group of animals showing significantly higher % inhibition levels at 13 and 15 months than at 12 months (*p* = 0.02 and *p* = 0.04 respectively). In order to confirm that these antibodies were virus-neutralising, the serum neutralising titre (SNT) was determined in all goats prior to vaccination (0 months), at 1 mpv and just before challenge (Fig. 3). For the whole group of animals challenged at 15 mpv, additional SNTs were determined at 12 and 13 months, which corresponds to before, and one month after, the booster vaccination for those animals given this second vaccination.

None of the sera from unvaccinated control goats had a detectable titre of PPRV-neutralising antibodies at any time, while the group of vaccinated goats had significantly higher PPRV-neutralising antibodies by 1 mpv (*p* < 0.0001) (Fig. 3a, b), with all but one animal having a detectable titre of such antibodies at this time point. All vaccinated animals had detectable PPRV-neutralising antibodies at the time of challenge. The overall level of neutralising antibodies in the animals vaccinated once, taken as a group, showed no significant change between 1 mpv and the respective time of challenge, including those challenged at 12 and 15 mpv. All goats receiving a booster vaccination demonstrated a significant increase in the titre of PPRV-neutralising antibodies after the booster vaccination, as seen by comparison of titres immediately prior to (12 months) and after (13 and 15 months) booster vaccination (*p* < 0.0001) (Fig. 3c); titres of PPRV-neutralising antibodies prior to booster vaccination were 1/16-1/96 and increased to at least 1/256 in all booster-vaccinated goats 1 month after the boost (Fig. 3b, c). No change was seen in the PPRV-neutralising titres of sera from the single-vaccinated animals between 12 and 15 mpv.

### Clinical response of animals to challenge with wild-type PPRV

**Fig. 4:**
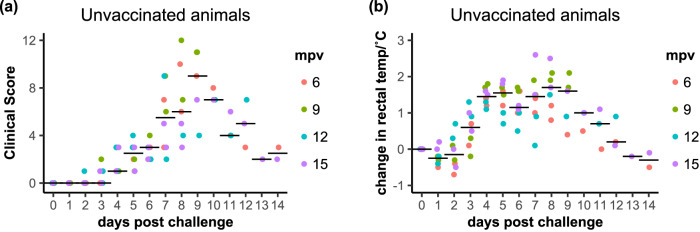
Clinical response of unvaccinated (control) animals to challenge with wild-type PPRV. **a** The total clinical score for each animal on each day of the challenge; (**b**) the daily rectal temperatures over the course of the challenge studies are shown as the change from the baseline temperature of the individual animal. **a**, **b** The data points for each animal are coloured according to the time post vaccination at which those animals were challenged. The position of the median value of all the samples at each time point is indicated by a horizontal bar.

**Fig. 5:**
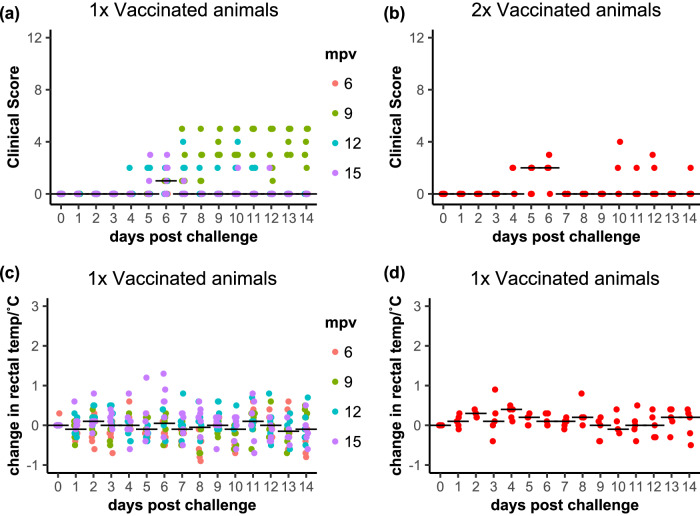
Clinical response of vaccinated animals to challenge with wild-type PPRV. **a**, **b** The total clinical score and (**c**, **d**) the change in rectal temperature from baseline over the course of the challenge studies are shown for (**a**, **c**) animals that were vaccinated once and (**b**, **d**) animals given an additional booster vaccination at 12 months. **a**, **c** The data points for each animal are coloured according to the time post vaccination at which those animals were challenged. **a**–**d** The position of the median value of all the samples at each time point is indicated by a horizontal bar.

In agreement with our preliminary testing of the stocks of challenge virus, the two unvaccinated goats (positive infection controls) in each of the four challenge studies showed clear clinical signs of PPR disease, including pyrexia, ocular and nasal discharge, lethargy, reduced or no food intake and diarrhoea, leading to high clinical scores (Fig. 4). In each of the 6 mpv and 15 mpv challenge studies, one unvaccinated control goat reached the humane endpoints of the study protocol at 8 days post challenge (dpc) and had to be euthanised, while in each of the 9 mpv and 12 mpv challenges both unvaccinated animals had to be euthanised early because they had reached the humane endpoints of the protocol, at 9 dpc in the 9 month study and at 7 and 12 dpc in the 12 month study. The other unvaccinated goats developed clear and significant disease, but not sufficiently severe to warrant early euthanasia. In our first study with recombinant adenovirus-based vaccine against PPRV, we used a single dose of 10^9^ infective units (IU) per animal, and observed complete protection against clinical disease^12^. Based on a second study in Kenya with a local breed of goats^14^, we deduced that 10^8^ IU was the minimum effective dose, and used that value for the vaccinations in this study. However, in the (significantly larger) Saanen goats used in this study, several vaccinated goats in each challenge group displayed mild and transient clinical signs (Fig. 5). No vaccinated animal had any detectable pyrexia (increase in rectal temperature of 1 °C or more) during the challenges at 6, 9 or 12 mpv, while at 15 mpv two single-vaccinated animals showed pyrexia for one day each. ANOVA analysis showed no difference in the pattern of rectal temperatures between the groups challenged at different times post vaccination (mpv was not a significant factor (*p* > 0.08), while vaccination status, dpc and the interaction term of these two factors were all significant (*p* < 0.0001)); the data from all four challenge groups were therefore analysed together. Unvaccinated animals as a group showed statistically significant elevation in rectal temperatures from 3 dpc to 10 dpc, while the groups of vaccinated animals (1x or 2x vaccinated) showed no significant change in rectal temperature at any time post challenge.

**Fig. 6:**
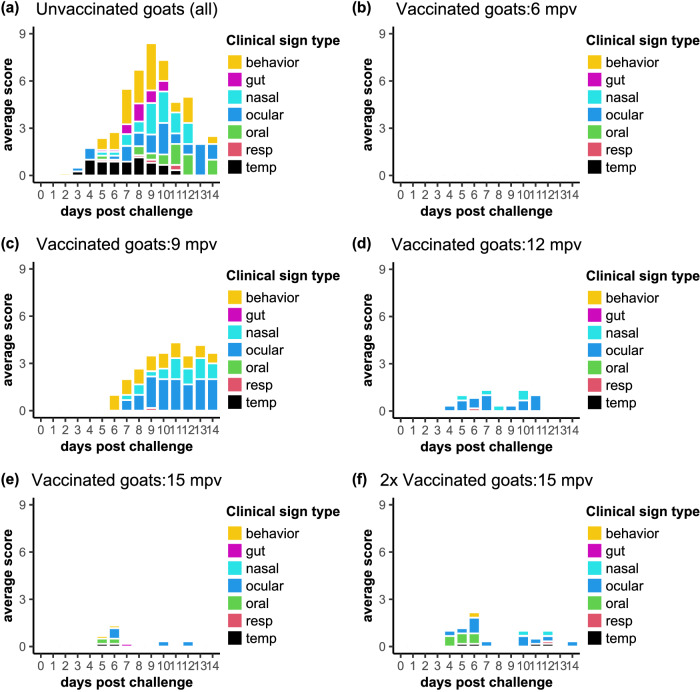
Breakdown of clinical scores by month and score type. The average clinical score of each type is plotted against day post challenge for (**a**) unvaccinated control goats, (**b**–**e**), single-vaccinated goats and (**f**) double-vaccinated goats. The 1x vaccinated animals at 6 mpv showed no clinical signs. The clinical signs are grouped by “behaviour” (movement, eating), “gut” (diarrhoea), “nasal” and “ocular” (primarily discharges from nose or eyes), “oral” (lesions in the gums or lips), “resp” (breathing difficulties or coughing) and “temp”, (significant changes of rectal temperature, included in this plot for completeness).

The clinical score data showed an unexpected result, with almost all the positive clinical scores for vaccinated animals appearing in the challenge at 9 mpv (Fig. 5a), while animals challenged at 6, 12 and 15 mpv showed very few clinical signs, with no individual animal showing any sustained clinical signs, and many animals showing no clinical signs at any stage during the challenge. It is difficult to explain this observation as a variation in the protection provided by the vaccine, since this would have required the immune protection to decrease at 9 mpv and then increase again at 12 mpv. It seems more likely that this observation resulted from an infection with another pathogen, unrecognised at the time, and perhaps acquired during animal transport from the vaccination facility in York to the high containment unit at Pirbright, an infection which led to clear, but not PPR-specific, clinical signs, primarily slight apathy and ocular and nasal discharge, but no pyrexia, oral lesions, respiratory symptoms or diarrhoea (see Fig. 6). There was no statistically significant difference between the clinical scores of the groups of vaccinated animals challenged at 6, 12 and 15 mpv (Figs. 5, 6), nor was there any overall difference between the single-vaccinated and double-vaccinated groups challenged at 15 mpv.

Looking at the responses in more detail, the clinical scores for the unvaccinated control animals were significantly above zero from 4 dpc to 13 dpc (Fig. 4). Combining all four challenge studies, there is a significant increase in the clinical score for the single-vaccinated animals on days 6-14 post challenge, although there is still a significant (*p* < 0.0001) difference between unvaccinated and vaccinated animals. If we omit the clinical scores for the 9 mpv challenge from the statistical analysis, including only the challenges at 6 mpv, 12 mpv and 15 mpv, then mpv is no longer a significant factor in the model, and the clinical scores of the 1x vaccinated animals showed no significant change from zero at any time during the challenge (Figs. 5, 6); the 2x vaccinated group showed a significant positive clinical score at 6 dpc (*p* = 0.0026), but not on any other day.

In the 15 months challenge study, one unvaccinated animal and six vaccinates across both vaccination groups developed small dry scabs (2–4 per animal, <3 mm in diameter) either in the nostril or on the lips between 3 and 6 dpi. It is not clear if these scabs were PPRV specific: such lesions have been described previously in PPR disease^16^, although not so early in the course of the disease. We have not previously observed them in our experimental PPRV infections, although we have not previously used goats of this age. Apart from this, and the previously mentioned peculiarities with the vaccinated animals at 9 mpv, the pattern of clinical signs seen during each challenge was the same.

### Detection of PPRV RNA in blood and ocular and nasal swabs following challenge with wild-type PPRV

EDTA blood, nasal and ocular swabs were collected every two days throughout each of the four challenge studies and processed for the detection of PPRV RNA by RT-qPCR.

**Fig. 7:**
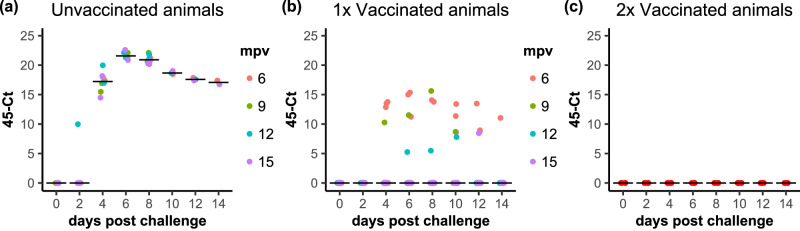
PPRV RNA detection by RT-qPCR in samples of whole blood from infected animals. The results of the PCR analysis are shown, plotted as 45-Ct, for (**a**) unvaccinated, (**b**) single-vaccinated and (**c**) double-vaccinated animals. **a**, **b** The data points for each animal are coloured according to the time post vaccination at which those animals were challenged. **a**–**c** The position of the median value of all the samples at each time point is indicated by a horizontal bar.

No difference was seen between the levels of viral RNA detected in the blood of unvaccinated animals challenged on different dates. As shown in Fig. 7a, PPRV RNA was detected in blood samples of all unvaccinated animals from 4 dpc, and levels of viral RNA continued high through to the end of the challenge period (14 dpc). In contrast, in the blood of the single-vaccinated animals, virus RNA was only detected irregularly and at levels close to the limit of detection of the assay. Curiously, two thirds of the positive blood samples from single-vaccinated animals (14 out of 22 samples) were in the group challenged at 6 mpv (Fig. 7b). When the levels of viral RNA in the blood are examined in detail over the course of the challenge, they were significantly different from zero in the unvaccinated animals at all times from 4 dpc to the end of the study (*p* < 0.0001, for all). In the single-vaccinated animals, the levels of viral RNA differ significantly from zero at 4, 6 and 10 dpc (*p* = 0.04, *p* = 0.009 and *p* = 0.047, respectively). However, this is entirely down to the positive samples seen at 6 mpv; if analysis is restricted only to the data from 9, 12 and 15 mpv, then the levels of RNA in the vaccinated animals do not differ from zero at any point in the challenge (*p* > 0.58).

None of the animals given the second vaccination had detectable viral RNA in their blood samples at any time post infection (Fig. 7c). Although no viral RNA was detected in blood from animals given the booster vaccination, and some samples from some single-vaccinated animals contained detectable viral RNA, the levels and frequency of detection were so low in all of the later groups (9, 12 and 15 mpv) that there was no statistically significant difference between single and double vaccination.

**Fig. 8:**
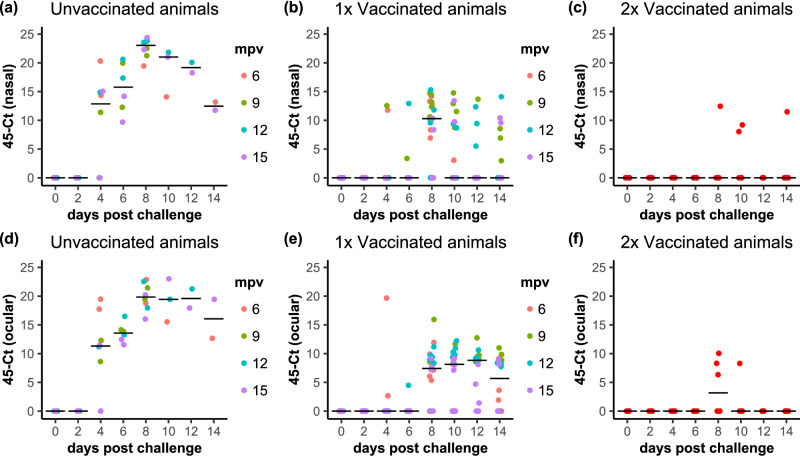
PPRV detection by RT-qPCR in nasal and ocular swabs from infected animals. The results of the PCR analysis are shown, plotted as 45-Ct; (**a**–**c**): nasal swabs, (**d**–**f**); ocular swabs. **a**, **d** Unvaccinated, (**b**, **e**) single-vaccinated and (**c**, **f**) double-vaccinated animals. (**a**, **b**, **d**, **e**) the data points for each animal are coloured according to the time post vaccination at which those animals were challenged. **a**–**f** The position of the median value of all the samples at each time point is indicated by a horizontal bar.

As found for the blood samples, the amounts of PPRV RNA in nasal and ocular swabs from the unvaccinated animals did not vary according to when the animals were challenged, and so the data from all these animals were considered as a group. PPRV RNA could be detected in the swabs from almost all unvaccinated animals by 4 dpc (Fig. 8a, d), and swabs were still positive at 14 dpc when the study terminated. Levels of viral RNA in nasal and ocular swabs for the whole group of unvaccinated goats were significantly above zero at all days from 4 dpc to 14 dpc. Virus isolation was carried out on positive samples at peak levels of viral RNA, and where there was sufficient fluid in the swab sample after RT-qPCR. Virus was isolated, usually at 8 dpc, from swabs from all unvaccinated animals, except for one such animal at 6 mpv (insufficient swab fluid for testing). Nasal swabs were, in this study, the best source of live virus, as infectious virus was successfully isolated from all seven unvaccinated animals tested, but only from an ocular swab from one of these animals.

PPRV RNA was detected more frequently in swab samples from vaccinated animals than in the blood samples, although the overall levels of RNA in both nasal and ocular swabs were still clearly different between vaccinated and unvaccinated animals (*p* < 0.0001 for both single- and double-vaccinated, for both kinds of swab). The difference in the viral RNA loads in the unvaccinated and single-vaccinated animals led to a difference in peak RT-qPCR Ct of 8-10 which, from the efficiency parameters of the real-time PCR used^17^ equates to a difference of 200-800-fold in numbers of copies of viral RNA. For the single vaccinates, the level of viral RNA in the nasal swabs differed significantly from background at 8 and 10 dpc, while for the ocular swabs the levels differed from background at 8, 10, 12 and 14 dpc. For the double vaccinates, although 5 out of the 6 animals showed at least one RT-qPCR positive swab on one day, the vast majority of the swabs, ocular or nasal, were negative, and the average for the group did not differ significantly from background on any day. Importantly, no infectious live virus was isolated from any of the swabs from vaccinated animals (single- or double-vaccinated) in any of the challenges.

### Induction of anti-PPRV N antibodies by challenge virus

Infection with PPRV leads to production of antibodies not only against the surface glycoprotein H but also the nucleocapsid protein N; cELISAs to detect PPRV-specific antibodies invariably look for anti-H antibodies^18–20^ or anti-N antibodies^21–23^. Anti-N antibodies are not virus neutralising, but are easily detected using a cELISA; anti-N antibodies are increased in animals infected with wild-type virus after vaccination with one of the live attenuated vaccine strains of PPRV^5^ and are produced in infected animals even when clinically protected by an H protein-based vaccine^11,12^, observations that are generally interpreted as indicating a low level of replication of a challenge virus even in animals protected from clinical disease. We assayed sera taken from all experimental animals in the study post challenge to measure the development of such anti-N antibodies, using the commercial anti-PPRV N protein cELISA.

**Fig. 9:**
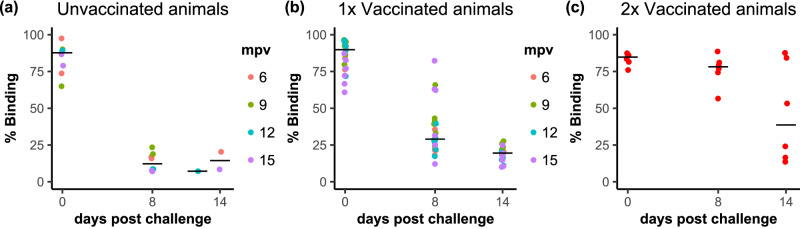
Development of anti-PPRV-N antibody in experimental goats following challenge. The result from the PPRV-N cELISA (% binding) for individual animals and days post challenge are shown for (**a**) unvaccinated, (**b**) single-vaccinated and (**c**) double-vaccinated animals. Note that, in this assay, the read out is the % binding of the control antibody, so high levels of anti-PPRV N antibody lead to low levels of % binding. **a**, **b** The data points for each animal are coloured according to the time post vaccination at which those animals were challenged. **a**–**c** The position of the median value of all the samples at each time point is indicated by a horizontal bar.

None of the goats had detectable anti-N antibodies prior to virus challenge (Fig. 9), confirming that the vaccination with rAdV5-H vaccine induced the production of anti-H specific antibodies only and therefore that a combination of anti-PPRV H and anti-PPRV N cELISAs can be used to differentiate between vaccinated and infected animals (DIVA capability). All eight unvaccinated goats had developed specific anti-PPRV-N antibodies by 8 dpc to the level that they were seropositive by the normal diagnostic test criterion (% binding <50%) (Fig. 9a). The development of anti-N antibodies at 8 dpc was also observed in the single-vaccinated animals, although only 20 out of 24 single-vaccinate animals reached the level of nominal seroconversion by 8 dpc (Fig. 9b). Taking the % binding value from the cELISA as a measure of the amount of anti-N antibodies in the sera, there was significantly less of such antibodies in the vaccinated animals relative to the unvaccinated (*p* = 0.007) at 8dpc, although this difference had disappeared by 14 dpc (Fig. 9a, b). These data confirm that some level of local virus replication led to the production of anti-N PPRV antibodies even in goats with no sign of PPRV RNA detectable in the blood, and are in agreement with our previous studies on goats vaccinated with rAdV5-H^12,14^.

The development of anti-N antibodies was significantly delayed in the booster-vaccinated group compared to the single-vaccinated animals (*p* < 0.0001), with no significant difference in the overall % binding for this group of animals between 0 and 8 dpc (*p* = 0.8868), and no sera reaching the level of nominal seroconversion by 8 dpc (Fig. 9c). By 14 dpc, the level of anti-N antibodies in the single-vaccinated animals was not significantly different to that in the unvaccinated, but the levels in the double-vaccinated animals were significantly lower than those in either the unvaccinated or the single-vaccinated. Three goats of the double-vaccinated group still had not seroconverted by 14 dpi (two goats remained seronegative and one was “inconclusive” (% binding between 50% and 60%)), suggesting that PPRV replication may have been further restricted in these animals.

## Discussion

Recombinant adenoviruses have been used as vaccine vectors for many years (see^24^ for a recent review of adenovirus-based vaccines), although it is only relatively recently that they have reached the stage of practical use as vaccines against human and animal diseases. Notably, several adenovirus-based vaccines were created to protect against SARS-CoV-2 during the recent global pandemic, including one based on an established chimpanzee adenovirus vaccine vector (ChAdOx1)^25^ marketed by Astra-Zeneca and one based on recombinant human adenovirus type 26 (rAdV26) marketed by Janssen^26^, as well as vaccines based on rAdV5^27^ or based on a heterologous prime-boost vaccine using rAdV5 and rAdV26 components^28^. In the field of livestock disease, a vaccine against foot-and-mouth disease virus (FMDV) based on rAdV5 has been conditionally licensed in the USA (see^29^ and references therein). While these and the many other adenovirus-based vaccine candidates that have been published have shown themselves to be immunogenic and, in some cases, protective against challenge with pathogenic virus, in most cases there is no information about the duration of immunity or protection. Even for the widely used vaccines against SARS-CoV-2, there are relatively few studies on the long-term maintenance of immunity, not least because of the difficulty of maintaining a cohort of subjects that have not been naturally exposed to the wild-type SARS-CoV-2 or received supplementary vaccinations with the same or alternative vaccines. The data that is available shows that, following immunisation with the ChAdOx1-based vaccine^30,31^ or one based on rAdV5^32^, antibody against the SARS-CoV-2 spike protein declines significantly over the following 12 months, with most of the decline in the first 6 months, corresponding to the known decline in protection provided by these vaccines. On the other hand, a study in cattle of the adenovirus-vectored vaccine against FMDV showed no significant loss of neutralising antibody over 9 months^29^. Similarly, a rAdV5 vectored vaccine against influenza A in swine induced an antibody response that was stable for up to 6 months^33^. In the study presented here, we show that the anti-PPRV antibody response to a single dose of rAdV5-H is stable for at least 15 months, whether this response is measured as virus neutralising antibody or as antibody binding to the PPRV H protein in a cELISA. Importantly, although the vaccine dose used was not sufficient to completely prevent viral RNA detection in nasal and ocular swabs and blood samples, and mild, transient clinical signs were observed in some of the vaccinated goats following virulent virus challenge, the level of protection against such challenge did not alter significantly over the 15 months of the trial. Given that the average age at slaughter of sheep and goats, at least for animals raised for meat in sub-Saharan Africa, is 15-20 months^34^, this suggests such a vaccine would be more than adequate for protecting animals destined for the meat trade, whether locally or internationally.

It is not clear why the vaccine against PPRV assessed in this study, like recombinant adenovirus-based vaccines against FMDV and swine influenza, gave stable protection, while those against SARS-CoV-2 showed a more rapid decline. It cannot be down to the virus vector backbone used, as the rAdV5-based vaccines against SARS-CoV-2 were similar to the ones based on ChAdOx1 in this respect. It is more likely that the duration of the antibody response depends at least in part on the specific antigen, irrespective of the vaccine vector, and therefore that the duration of protection will have to be evaluated individually for every antigen. What we have established in this study is that this type of vaccine, expressing the PPRV H glycoprotein, is capable of eliciting a long-lasting protective immune response.

This study has also demonstrated that a homologous booster vaccination was effective at increasing PPRV-specific antibodies. This observation is in accord with the findings from the use of adenovirus-based vaccines against SARS-CoV-2, where a second vaccination with the same vaccine was shown to increase anti-SARS-CoV-2 antibody in the case of the vaccine based on ChAdOx1^35^ and probably reflects the fact that gene expression from the non-replicating adenoviruses is dominated by the inserted transgene^36^, although it is notable that homologous boosting with the rAdV26 vaccine against SARS-CoV-2 had little effect on spike antibody levels^37^.

While the booster vaccination reduced the levels of viral RNA in the blood and swabs in our experimental animals, and clearly decreased the replication of the challenge virus, as shown by the reduction in the levels of anti-N antibody, it was not enough to completely eliminate challenge virus replication or clinical signs. In fact our observations in this study are very similar to those of Rojas et al.^11^ who used two doses of 10^8^ IU of a very similar vaccine in young sheep and also detected PPRV RNA in the blood of vaccinated animals, also at >100-fold lower levels than seen in unvaccinated animals. Rojas et al also observed strongly reduced or absent clinical signs, although the challenge virus clearly replicated in their vaccinated animals, as shown by the development of anti-PPRV N antibodies after challenge. As we also observed in this study, despite the detectable levels of intermittent viral RNA in blood samples or, in our case, ocular and nasal swabs, there was no detectable excretion of live virus. It is possible that very low levels of live virus were being excreted which were not detected by our virus isolation protocol. In future studies, it would be interesting to introduce naïve animals to the vaccinated animals 2-3 days after challenge, this providing the ultimate test for effective excretion of live virus. It is not possible to say from the study presented here whether the absence of live excreted virus was due to the pre-neutralisation of virus particles by circulating anti-H antibody, or to an absence of whole virus particles, with the detected viral RNA reflecting cell debris from infected cells. There is probably a minimum level of virus that must be circulating in infected animals in order for live virus to be excreted: a recent study^38^ using modern RT-qPCR detection methods have detected viral RNA in blood and swabs of animals following vaccination with the live attenuated Nigeria/75 vaccine strain of PPRV, at similar levels to that found in this study in single-vaccinated animals following challenge, even though the live attenuated PPRV vaccine strain has never been reported as being excreted.

While the dose of vaccine used in this study was sufficient to suppress challenge virus replication in a large-scale trial conducted in Kenya^14^, it would appear that a significantly higher dose of vaccine than we inferred previously is required to completely protect animals from all clinical signs, and doses of 10^9^ IU per animal, previously shown to give complete clinical protection^12^, would be recommended for field use of such a vaccine. The reason for our previous underestimate of the minimum protective dose is probably the low pathogenicity of the challenge virus used in the goats in the Kenya study. Clearly, a generally protective dose of vaccine must protect from disease caused by the most pathogenic strain, one which causes unprotected goats to develop severe clinical signs. In the original test of the vaccine, where the vaccination dose was 10^9^ IU per animal, the unvaccinated animals developed severe disease, with 3 out of 4 unvaccinated goats having to be euthanised at 10 dpc^12^, while the vaccinated animals showed no clinical signs, and therefore 10^9^ IU per animal should be taken as the actual fully protective dose. The cost of this kind of vaccine is therefore going to be significantly higher than the cost of the existing live attenuated PPRV vaccines. However, the currently-used attenuated PPRV vaccines will be more than adequate for the large-scale vaccination required during the main stages of the eradication campaign, and vaccines such as rAdV5-H will come into their own when a DIVA capability and/or the avoidance of live PPRV become critical factors. In addition, for field delivery of PPR vaccines, the cost of the actual vaccine is a small fraction (10%-15%) of the total cost of vaccination (e.g^39,40^.), so a 10-fold increase in the cost of the vaccine would only lead to an approximate doubling of the total cost of vaccination. The important finding from the study reported here is that this kind of vaccine can elicit very durable protection and a persistent antibody signature, making it suitable for use as a DIVA vaccine in the field.

The other change in the commercial preparation of this kind of vaccine that will be required will be in the cell line used for the amplification of vaccine stocks. The stocks used in this study and our previous studies were grown in HEK293 cells, in which recombination between the adenovirus vector and adenovirus DNA integrated into the HEK293 genome, even though rare, can lead to the production of replication competent adenoviruses. Significant effort was applied to ensure that the stocks used in the study here were free of such recombinants. Commercial production of this vaccine would require production in one of the cell lines such as PER.C6^41^ that have no overlap between the adenoviral sequences in the vector and those in the helper cell line, in order to prevent creation of replication competent adenoviruses which may pose a risk to people administering the vaccine.

In summary, we have developed a vaccine against PPR that provides the capability of a DIVA test and shown here that the protection provided by that vaccine, and the antibody signature that provides that DIVA capability, are both stable for at least 15 months.

## Methods

### Virus stocks

All virus stocks were cultured in Vero cells expressing canine signalling lymphocyte activation marker (SLAM), the morbillivirus receptor (Vero-Dog-SLAM; VDS)^42^. Wild-type isolates of PPRV used in this study were: PPRV/Cote d’Ivoire/1989 (CdI) (lineage 1), PPRV/Ghana/Accra/1978 (Ghana) (lineage 2) and PPRV/Georgia/Tbilisi/2016 (Georgia) (lineage 4), all of which were from the Pirbright Institute virus archive, and PPRV/Iraq/2011 (Iraq) (lineage 4) (originally called PPRV/Kurdistan/2011^43^), the kind gift of Dr Bernd Hoffman, FLI, Germany. All four strains may be obtained through the European Virus Archive-GLOBAL (EVAg; www.european-virus-archive.com). Full or almost full genome sequences of the virus strains are available in GenBank: CdI, OL741724; Ghana, OR286502; Georgia, MF737202; Iraq, MK408669. Virus stocks were grown and titrated on VDS as previously described^15^. The passage history (from last passage in goats) of the stocks used to infect animals were: CdI (VDS1); Ghana (primary lamb kidney cells (LK)1, Vero1, VDS1); Georgia (VDS2); Iraq (CV1-goat SLAM1, VDS3).

### Vaccine

Stocks of recombinant adenovirus expressing PPRV H protein were produced by the Viral Vector Core Facility, Pandemic Sciences Institute, Oxford, using an E1, E3 deleted human adenovirus type 5 vector (Oxford University). In order to be certain that the preparations of vaccine were safe to use at BSL1, all preparations of vaccine were screened for the recovery of replication competency, which can occur if there is recombination between the E1, E3-deleted vaccine vector and the 11% fraction of the adenovirus genome which is contained in the HEK293 cells used to propagate the vaccine. All vaccine preparations were tested by several methods: (i) PCRs to test the vaccine preparations for the presence of the E1A gene and whether this E1A gene was integrated into the vaccine vector, which would show that recombination events had occurred during vaccine propagation in the HEK293 cells; (ii) serial passage on permissive, but non-complementing cells (in this case, HeLa cells), checking for vector replication at each passage by (a) visual observation for the appearance of cytopathic effect (cpe) and (b) PCR for the detection of amplification of the adenoviral E1A gene, which would test for the creation of a replication-competent virus. Validation studies using a preparation of an irrelevant recombinant adenovirus (expressing GFP) did show cpe at the second passage level in HeLa cells, and also E1A gene in the DNA extracted from the cells at the first passage, showing that the assays used are able to detect replication-competent viruses in such vaccines.

All vaccine preparations showed cpe on inoculation of one or two full doses (1–2 × 10^8^ infective units (IU)) into HeLa cells in 25 cm^2^ flasks (multiplicity of infection (m.o.i.) ~33 or ~77) or 75 cm^2^ flasks (m.o.i. ~11), but this cpe was not observed at the second or third passage for the rAdV5-H vaccine preparations. PCR detected E1A gene, integrated into the vaccine vector, in the rAdV5-H vaccine preparations at low copy number (calculated as 0.8-40 copies per vaccine dose), but the E1A gene was not detectable in DNA extracted from the inoculated HeLa cells at any passage, showing that, although a low level of recombination events had occurred during vaccine manufacture, no actual replication-competent virus had been generated.

### Immunological and other assays

Assays using competition ELISAs (cELISAs) specific for antibodies against the PPRV H (in-house) and N protein (ID Screen PPR Competition ELISA, ID Vet Garbles, France), and determination of the serum neutralisation titre (SNT) against PPRV were all carried out as previously described^5^. Automated extraction of RNA was performed using 100 μl of sample (EDTA blood, cell culture isolates, tissue extracts, ocular or nasal swabs) using the MagVet Universal nucleic acid extraction kit (Thermo Fisher Scientific) on a Kingfisher Flex automated extraction platform (Thermo Fisher Scientific). RNA was eluted into 80 μl of elution buffer and was stored at 4 °C prior to analysis. The PPRV real-time RT-qPCR assay was performed using the Express One-Step Superscript qRT-PCR kit (Life Technologies) as previously described^17,44^.

### Virus isolation

Virus isolation was attempted using blood and swab samples from vaccinated and unvaccinated goats that were positive in PPRV RT-qPCR. Samples were inoculated onto 80% confluent monolayers of VDS cells in the wells of 6-well plates. The cells were cultured in DMEM supplemented with HEPES, 100 U/ml penicillin, 100 µg/ml streptomycin, 2mM L-glutamine and 0.1 mg/ml zeocin and containing 10% heat-inactivated FBS. The cells were then incubated for 2 h in a humidified chamber at 37 °C to allow for virus adsorption, then the inocula were removed and the monolayer washed once with medium before adding fresh medium and incubating as normal for 7 days. The cell monolayers were then frozen at −80 °C, thawed, and cell debris removed by centrifugation at 1000 × *g* for 5 min. The supernatants were passaged on VDS cells for a further 7 days before freeze-thaw lysis as above. Virus isolation and propagation was confirmed by a positive PPRV RT-qPCR test on the supernatant from the second passage.

### Animal experiment 1: preliminary test of wild-type PPRV stocks

Stocks of the four different strains of PPRV isolates were tested to establish and confirm the best challenge virus leading to typical and explicit PPR clinical disease when used to infect Saanen breed goats. For each strain, 3 male animals (9-15 months old) were infected intranasally with 1 × 10^5^ TCID_50_ of PPRV in a volume of 1 ml using a syringe coupled to a mucosal atomisation device (MAD Nasal™, Teleflex Inc, USA). Infected animals were monitored twice daily. Rectal temperatures were measured and clinical signs assessed daily. Blood samples (EDTA tube), ocular swabs and nasal swabs were taken on days 0, 2, 4, 6, 8 and (for the animals challenged with Iraq) 9 days post challenge (dpc). Blood for serum was collected on day 0, day 8 and/or on the day the animals were euthanised. For this study, animals were euthanised when it was clear that the PPRV strain had elicited overt PPR-specific clinical disease, which was considered the scientific endpoint.

### Animal experiment 2: vaccine study

Thirty-eight Saanen breed goats (male, 10 months old at time of vaccination), were group housed in secure indoor enclosures and outdoor paddocks at APHA/FERA Sand Hutton National Agri-Food Innovation Campus, Sand Hutton, York. Each goat was randomly assigned to either the vaccinated or unvaccinated groups. Thirty animals were vaccinated intra-muscularly with 1 ml PBS containing 10^8^ IU of the previously described^12^ recombinant human adenovirus vaccine expressing the H glycoprotein of PPRV/Nigeria/71 vaccine strain. Eight additional animals, co-housed with the vaccinated animals, were mock vaccinated using 1 ml PBS injected intra-muscularly (unvaccinated group) to act as controls during the subsequent challenges. Animals were repeatedly tested for caseous lymphadenitis by ELISA, with Western blot confirmation as required, during the course of the study, due to the prevalence of this disease in UK goats. Blood for serum was collected before vaccination and at 1, 3, 6, 9, 12, 13 and 15 months after the initial vaccination (10 ml blood taken by vacutainer from one of the jugular veins, using alternate sides of the neck from each bleed). At 6, 9 and 12 mpv, groups of six vaccinates and two unvaccinated control animals were transferred to the high-containment units at the Pirbright Institute and infected intranasally with 10^5^ TCID_50_ PPRV Ghana. At 12 mpv, 6 vaccinates were given a further identical dose of vaccine. At 15 mpv, these animals, along with the remaining 6 single-vaccinated animals and two controls were transferred to the Pirbright Institute and challenged as above with PPRV Ghana strain. During the 15 mpv challenge, one of the double-vaccinated animals suffered a broken horn while fighting with another animal, and had to be treated with antibiotics. This animal also developed unique clinical signs, including a rise in rectal temperature at 11 days post infection. Since it was possible this animal had suffered additional stress and/or infection through the head wound, this animal was omitted from the part of the study that measured the response to challenge with wild-type virus.

All animals infected with PPRV were monitored twice daily. Rectal temperatures were measured, and clinical signs scored, daily. EDTA blood, nasal and ocular swabs were taken on days 0, 2, 4, 6, 8, 12 and 14 dpc (for the first challenge, at 6 mpv, swabs were only taken at 0, 4, 8, 10 and 14 dpc). Blood for serum was taken at 0, 8 and 14 dpc. Where any animal had to be euthanised due to meeting the humane endpoints defined for the study, EDTA blood and blood for serum were collected on the day of euthanasia, even if not scheduled.

All animals were euthanised by an overdose of Pentobarbital (Dolethal, Vetoquinol). Animal studies were carried out in accordance with the UK Animal Scientific Procedure Act (ASPA) 1986 under the UK home office project licence 70/8833. All animal studies were reviewed and approved by the local Ethical Review Boards at the Pirbright Institute and at APHA/FERA Sand Hutton.

### Clinical scoring

Clinical scoring was carried out essentially as previously described^5^ with the exception that ocular and nasal congestion, or congestion of the gums, was not considered, as we found it impossible to reliably identify disease-induced mild congestion distinct from other environmental effects in our experimental animals. We also expanded the defined conditions for more severe, and therefore higher scoring, clinical signs. The final clinical scoring system used was therefore: a score of 1 was given for rectal temperatures 1 °C to 2 °C above baseline for the animal, and a score of 2 was given for temperatures >2 °C above baseline. Similarly, a score of 1 was given for reduced eating, a soft stool, a repeated cough, or mildly apathetic behaviour; a score of 2 was given for visible ocular or nasal discharge, one to two lesions in the gums, diarrhoea, or reluctance to walk or stand; and a score of 3 was given for difficulty in breathing, ocular or nasal discharge sufficient to cause distress, necrotic oral lesions, a refusal to eat, remaining recumbent when approached or isolated from other animals, or bloody/watery diarrhoea. Animals reaching defined humane endpoints of a moderate severity were euthanised humanely (Supplementary Table 1). Note that the full cumulative score was used for defining humane endpoints but, in order to avoid using and plotting the same data twice, the clinical score used for statistical analysis and displayed on graphs omitted the contribution from rectal temperatures, which were analysed separately.

### Statistical analyses and graphs

All datapoints represent distinct samples. All statistical analyses were carried out using R^45^. For all animal studies, the data were analysed using linear mixed models (package *nlme*^46^) in which vaccination status and time (either months post vaccination or days post challenge, or both) were fixed factors and animals were random factors. Comparisons between groups and correction for multiple comparisons were carried out using *emmeans*^47^. All *p* values given in the text are either from analysis of the linear mixed models using the R function *anova* (F statistic) or from two-tailed comparisons carried out with *emmeans* (t statistic with either “tukey” correction for simple pairwise multiple comparisons or “mvt” correction for other cases). In order to improve the normality of the data, SNT data were analysed as log(titre+1). Animal rectal temperatures were analysed as the deviation from the baseline temperature for each animal. All graphs were created in R using the package *ggplot2*^48^.

### Reporting summary

Further information on research design is available in the Nature Research Reporting Summary linked to this article.

### Supplementary information

Supplementary Information

Reporting summary

## Data Availability

The relevant data that support the findings of this study are available from the corresponding author upon reasonable request.
