# Supplementary material for: Long-term trial of protection provided by adenovirus-vectored vaccine expressing the PPRV H protein
Source: NPJ Vaccines. 2024 Jun 3;9:98. doi: 10.1038/s41541-024-00892-2 (PMC11148195; doi:10.1038/s41541-024-00892-2)
Supplement: Supplementary file 1 — Supplementary Information [file 41541_2024_892_MOESM1_ESM.pdf]

Supplementary Table 1. Clinical scoring and humane endpoint table for post-vaccination PPRV challenge studies

The table shows the scores given to different severity levels of each category of clinical sign, which severity levels are considered when judging the humane endpoint (marked in red), and how long any one of these levels of clinical sign may continue before the endpoint is declared to have been reached. In addition, if two or more red severity levels are present on the same day, the humane endpoint is declared to have been reached.

**Clinical Sign Category**	**Subcategories(score)[days[1]]**		
**Rectal temperature[2]**	Baseline[3]+1°C to baseline+2°C (1)	>Baseline+2°C (2) [3]	
**Feeding**	Reduced eating (1)	Only eating hay/straw (2)	Not eating (3) [2]
**Behaviour**	Quiet/slow (1)	Lethargic (2)	Remains incumbent when approached or touched, head hung, back arched or isolated from group (3) [1]
**Ocular**	Discharge (2)	Discharge sufficient to cause distress (3) [1]	
**Nasal**	Discharge (2)	Discharge sufficient to cause distress (3) [1]	
**Oral**	Gum lesions (2)	Necrotic gum lesions (3) (In red if >1 necrotic lesion >3mm in diameter [1])	
**Respiratory**	Repeated cough (1)	Persistent coughing (2) [2]	Laboured/increased breathing (3) [1]
**Digestion**	Loose faeces (1)	Diarrhoea (2)	Bloody/watery diarrhoea (3) [1]

[1]If only one red clinical sign is seen, the endpoint is reached when this number of consecutive days with the sign is reached

[2]The scores attributed to the rectal temperatures were not included in the "Clinical score" used in this paper since the rectal temperature was analysed separately.

[3]Baseline rectal temperature for each animal is determined from measurements taken on at least three days before challenge infection.
